# A non-destructive image-based approach to quantify blood meal size in *Lutzomyia longipalpis* (Diptera: Psychodidae)

**DOI:** 10.1590/0074-02760250158

**Published:** 2026-05-01

**Authors:** Lidiane Medeiros da Costa, Maurício Roberto Viana Sant’Anna, Laura Aquino Generoso, Geisler Peixoto da Cruz, Grasielle Caldas D’Ávila Pessoa, Ricardo Nascimento Araujo, Nelder de Figueiredo Gontijo, Marcos Horacio Pereira

**Affiliations:** 1 Universidade Federal de Minas Gerais, Instituto de Ciências Biológicas, Departamento de Parasitologia, Laboratório de Fisiologia de Insetos Hematófagos, Belo Horizonte, MG, Brasil; 2 Pontifícia Universidade Católica de Minas Gerais, Belo Horizonte, MG, Brasil; 3 Universidade Federal de Minas Gerais, Instituto de Ciências Biológicas, Departamento de Parasitologia, Laboratório de Entomologia Médica, Belo Horizonte, MG, Brasil; 4 Universidade Federal de Minas Gerais, Instituto de Ciências Biológicas, Departamento de Parasitologia, Laboratório de Artrópodes Hematófagos, Belo Horizonte, MG, Brasil

**Keywords:** Lutzomyia longipalpis, blood meal size, image analysis, haemoglobin quantification

## Abstract

**BACKGROUND:**

Phlebotomine sand flies are hematophagous vectors of major human pathogens, including *Leishmania* spp., with blood ingestion essential for reproduction and vector competence. Accurate quantification of blood meal volume is crucial for understanding physiological processes and transmission dynamics.

**OBJECTIVES:**

Here, we introduce a novel, non-destructive image-based method to estimate blood intake in *Lutzomyia longipalpis*, the principal vector of *Leishmania infantum* in the Americas.

**METHODS:**

High-resolution images of unfed and blood-fed females were analysed using Fiji ImageJ (open-source software) when several morphometric parameters were measured and validated against biochemical haemoglobin (Hb) quantification.

**FINDINGS:**

Blood-fed females exhibited a 56.6% increase in abdominal width and a shift toward a rounded body shape, which was strongly correlated with a visible transilluminated abdominal area (R² = 0.92). Some parameters, such as mean grey value and abdominal length, showed a low to moderate correlation with Hb content (R < 0.60). However, the correlation with abdominal area and width was R ≈ 0.90, indicating those are reliable parameters that can be used to estimate blood intake by *Lu. longipalpis* females.

**MAIN CONCLUSIONS:**

Unlike spectrophotometric methods, this approach preserves specimen integrity, which, in theory, enables longitudinal studies on physiology and host-parasite interactions. This methodology offers a reliable, scalable, and cost-effective alternative for estimating blood meal.

Phlebotomine sand flies are haematophagous dipterans belonging to the Family Psychodidae. More than 900 species have been identified, with a global distribution that excludes only Antarctica. The greatest abundance and diversity occur in tropical and subtropical regions.^1^ Certain phlebotomine species act as vectors of pathogens responsible for viral (*Phlebovirus* spp.), bacterial (*Bartonella* spp.), and protozoan (*Leishmania* spp.) infections in both humans and animals.^2^ ^,^ ^3^ ^,^ ^4^ ^,^ ^5^ Leishmaniasis remains an important global health issue, with over 272,000 new human cases reported in 2023.^6^ *Lutzomyia longipalpis* is recognised as the principal vector of *Leishmania infantum* in the Americas, the causative agent of American visceral leishmaniasis (AVL).^7^ Notably, *Lu. longipalpis* exhibits a high degree of adaptation to urban environments, with multiple studies indicating a substantial expansion of its urban distribution since the 1980s, which has significantly altered the transmission dynamics and epidemiological patterns of visceral leishmaniasis.^8^ Moreover, increasing molecular and ecological evidence supports the hypothesis that *Lu. longipalpis* comprises a species complex of cryptic taxa with subtle morphological similarities, potentially accounting for variations in vectorial capacity across distinct geographic populations.^7^ ^,^ ^9^ ^,^ ^10^ ^,^ ^11^

In phlebotomine sand flies, adult males and females feed on carbohydrate-rich sources such as nectar, plant sap, and aphid secretions. Carbohydrates are initially directed to the crop in the anterior midgut, where they are stored and later transferred to the midgut for digestion.^12^ Blood is obtained through telmophagy, where superficial dermal layers are disrupted using short, rigid mouthparts to create blood pools that are ingested.^13^ This feeding method facilitates ingesting *Leishmania* parasites in the skin.^14^ Under laboratory conditions, blood meals typically last 1-5 min, during which females may ingest an amount of blood approximately equivalent to their body weight (0.1-0.6 mg).^15^ Blood is directed to the midgut, leading to abdominal distension mediated by the pleural membranes.^16^ Blood digestion takes 48-72 h. Digestion products such as amino acids, fatty acids, and sugars are absorbed by enterocytes and transported to support ovarian maturation.^17^ ^,^ ^18^ ^,^ ^19^ ^,^ ^20^ The number of eggs produced is proportional to the volume of blood ingested, with a maximum determined by the number of ovarioles (approximately 50).^21^ ^,^ ^22^ ^,^ ^23^ In *Lu. longipalpis*, females are anautogenous, exhibiting gonotrophic concordance, which means they lay eggs only after a blood meal during their first reproductive cycle.^24^ Typically, 30-70 eggs are laid per gonotrophic cycle. Beyond reproductive consequences, the volume of blood meal can influence the efficiency of pathogen transmission.^25^ Therefore, quantifying blood intake is crucial for understanding vector biology, population dynamics, and disease epidemiology, as well as for developing effective control strategies.^26^ ^,^ ^27^

Several methods have been employed to estimate blood meal volume, including gravimetric analysis,^17^ ^-^ ^27^ radioisotope labelling,^28^ vital dyes,^29^ and spectrophotometric haemoglobin (Hb) quantification.^27^ ^,^ ^30^ ^,^ ^31^ ^,^ ^32^ ^,^ ^33^ For instance, Volfová^27^ correlated Hb dosage with body weight gain in ten sand fly species fed on various hosts. Drabkin’s solution, containing cyanide and ferricyanide, is widely used to form a stable coloured complex with Hb for quantification.^34^ ^,^ ^35^ ^,^ ^36^ Martin-Martin^33^ employed this reagent to estimate blood volumes in *Lu. longipalpis* in a study assessing the immunogenicity of salivary proteins and their impact on *L. major* infection. While effective, many existing techniques for estimating blood meal size in sand flies are invasive, destructive, or chemically dependent, limiting their applicability in live-insect studies. In response, we propose an innovative, non-destructive, and chemical-free image-based method suitable for live and anesthetised female sand flies. This method, which involves capturing high-resolution images of females after blood feeding on anesthetised hamsters and analysing them using Fiji ImageJ (open-source software),^37^ offers a promising alternative to existing techniques in insect research.

## MATERIALS AND METHODS

*Sand fly rearing* - *Lu. longipalpis* specimens originating from Teresina (Piauí, Brazil) were used in this study. Sand flies were maintained in a closed colony at the insectary of the Laboratory of Haematophagous Insect Physiology (ICB-UFMG) under controlled temperature conditions (25 ± 1ºC), according to the protocol described by Modi and Tesh.^38^ Adults were fed ad libitum with a 15% sucrose solution on cotton pads and kept under a 12:12 h light/dark photoperiod. Blood feeding was performed weekly using hamsters (*Mesocricetus auratus*), aged four-eight weeks and weighing between 100 and 150 g. The animals were anaesthetised intraperitoneally with sodium thiopental (Thiopentax®, Cristália, Brazil), prepared at a concentration of 50 mg/mL and administered at a dose of 100 μL per 100 g of body weight (50 mg/kg). All procedures followed the guidelines established by the Animal Ethics Committee of the Federal University of Minas Gerais (CEUA protocol 262/2021).

*Feeding assay* - For experimental procedures, females aged five-seven days post-emergence were selected. Insects were divided into two groups (n = 60 females and 10 males per group) and housed in plastic containers (11 cm height, 8.5 cm diameter) covered with mesh fabric lids. The sugar solution was removed 12 h before the assay. In the control group (CG), females were maintained under fasting conditions, while in the experimental group (EG), females were allowed to feed on hamster for 1 h. The hamster used for blood feeding had no prior contact with sand flies or anaesthetic agents.

*Image acquisition and analysis* - To compare meal size estimation between image analysis and Hb quantification, insects from both groups were euthanised immediately after the feeding assays by freezing at -20ºC for 10 min. Females were subsequently positioned in lateral decubitus on a Petri dish containing graph paper as a size reference. The plate was positioned on a transilluminator with fixed bottom illumination, at a distance of 6.3 cm from the Casio Ex-FH20 camera. Images were captured at a resolution of 3456 × 2592 pixels with an exposure compensation of +2 exposure value (+2 EV). White balance and ISO settings were kept in automatic mode. Captured images were processed and analysed using Fiji ImageJ software (https://imagej.net/) (version 1.53f51). Image parameters related to size, shape, and colour were obtained by selecting the following features using the “Set Measurements” tool: area; shape descriptors (circularity and roundness); integrated density; mean grey value; perimeter and fitting an ellipse. To improve visualisation of small amounts of ingested blood in females, the pseudo flat-field correction tool was used, which allows background resulting from uneven illumination to be subtracted from both greyscale and true-colour images. The graphical representations of the data were produced using GraphPad Prism 10.6.1 software.

**Fig. 1:**
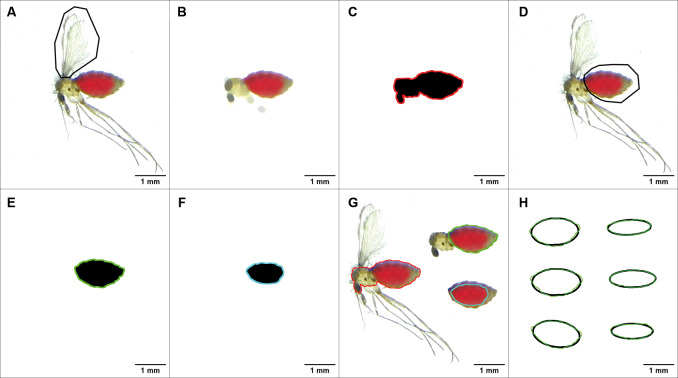
steps for obtaining the regions of interest (ROIs) in the body of *Lutzomyia longipalpis*. (A-C) Selection of the contour of the entire body (TBS) excluding appendages; (D-E) selection of the abdominal contour (ABS); (F) selection of the contour of the red-coloured abdominal region (RCS) in blood-fed females; (G) localisation of the ROIs (TBS, ABS, and RCS) relative to the body parts/regions of a blood-engorged female sand fly; (H) comparison between the contours of the ABS ROIs (green lines) of blood-fed (left) and non-blood-fed (right) females with the corresponding ellipses generated by the “Fit Ellipse” tool (black lines). The Roman numerals I and II represent the major (~length) and minor (~width) axes.

*Selection of regions of interest (ROIs)* - Following image calibration (histogram and scale adjustment), processing was performed (math + max 200 + brightness/contrast adjustments) to delineate ROIs. Image segmentation tools (thresholding or colour thresholding), which use grey-level intensity (8-bit images) or colour information from red, green and blue (RGB) images to separate the object of interest from the background, were applied. Three ROIs were selected [Supplementary data (Figure)]: the whole body excluding appendages (TBS); the abdominal region alone (ABS); the red-coloured abdominal region corresponding to the ingested blood volume visible through cuticle transparency (RCS). A standardised protocol was followed to define the ROIs, as illustrated in Fig. 1. For TBS: wings were removed (polygon selection + clear), a top-hat filter was applied to eliminate bristles and appendages, and the body contour was defined (conversion to 8-bit image + threshold + wand tracing tool) (Fig. 1A-C). For ABS: the abdomen was isolated from the thorax (polygon selection + clear outside), bristles were removed or attenuated (median filter), and the abdominal contour was delineated (8-bit conversion + threshold + wand tracing tool) (Fig. 1D-E). For RCS: the red-coloured area within ABS was selected using the colour threshold, clear outside and wand tracing tool (Fig. 1F). After selection, ROIs were compared with the original RGB images, corrected when necessary, and saved.

*Hb quantification* - To estimate the amount of blood ingested by the sand flies, Hb quantification was performed using Drabkin’s reagent, following the manufacturer’s instructions (Labtest Diagnóstica S.A). Each insect from the control and experimental groups was placed in a 2 mL Eppendorf tube containing 20 µL of Drabkin’s reagent and labelled with the same identification number used in the captured images. The samples were then homogenised using a micro-homogeniser. After homogenisation, an additional 380 µL of Drabkin’s reagent was added to each tube, followed by centrifugation at 10,000g for 5 min. Subsequently, 200 μL of the supernatant was transferred to a well of a 96-well microplate. The standard curve was generated by performing a two-fold serial dilution of heparinised human blood (0.2 µL/mL) across 12 wells of the microplate containing Drabkin’s reagent (final volume of 200 μL), corresponding to a range of 5.0 to 0.002 µL of blood. After preparation, the optical density (OD) of the resulting solution was measured at 540 nm.

*Statistical analysis* - Data were analysed using GraphPad Prism 10.6.1 software. The Kolmogorov-Smirnov test assessed data normality. The t-test was used to test variables with a normal distribution. In cases where variables did not present a normal distribution, analyses were performed using the Mann-Whitney test. P < 0.05 was considered significant. The degree of association and the relationship between parameters obtained from image analysis and those from the Hb OD at 540 nm were assessed using Pearson correlation tests. Observations with absolute studentised residuals > 3 and a Cook’s distances > 4/n were considered outliers,^39^ and the linear regressions and R² values were re-estimated after excluding outliers from the dataset.

## RESULTS

**Fig. 2:**
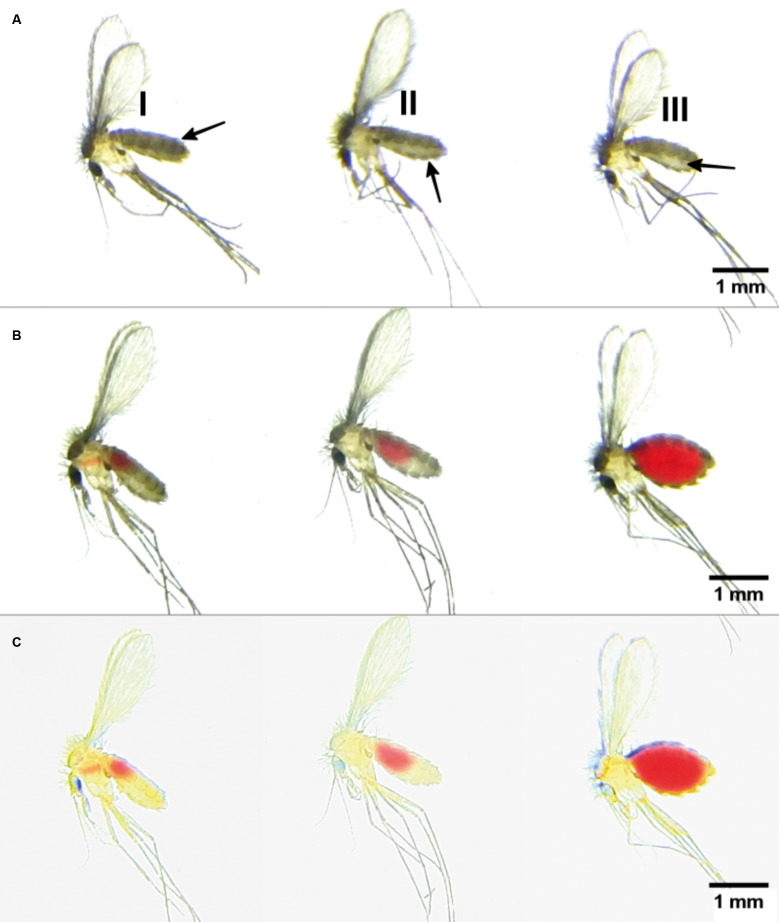
original red, green and blue (RGB) images of three unfed *Lutzomyia longipalpis* females (control group, A) and three females that fed on hamsters (experimental group, B-C). The same blood-fed females after pseudo-flat field correction (BioVoxxel), highlighting red coloration in the digestive tract. Arrows indicate tergal plates (I), sternal plates (II), and pleural membrane (III).

*Image-based assessment of blood feeding in CG and EG females* - Lateral-view images of *Lu. longipalpis* females enabled clear visual differentiation between unfed and blood-fed individuals. Unfed females (CG group) exhibited variable abdominal profiles: some showed collapsed abdomens, where only tergal plates were discernible with poorly defined external plates and no visible pleural membrane (Fig. 2AI), while others displayed moderately expanded abdomens, characterised by a distinct separation between tergal and external plates via a visible pleural membrane spanning all abdominal segments (Fig. 2AII-AIII). In blood-fed females (EG), the most remarkable abdominal expansion consistently occurred between the fourth and fifth abdominal segments (Fig. 2B).

The use of transmitted light during image acquisition facilitated the detection of reddish coloration in the thoracic and abdominal midgut (Fig. 2B), indicative of blood ingestion. Even females with minimal blood intake and no remarkable abdominal distension were reliably identified by applying image brightness and contrast adjustments (Fig. 2C). All females exposed to the host (EG) showed evidence of blood ingestion, as confirmed by image-based analysis.

**Fig. 3:**
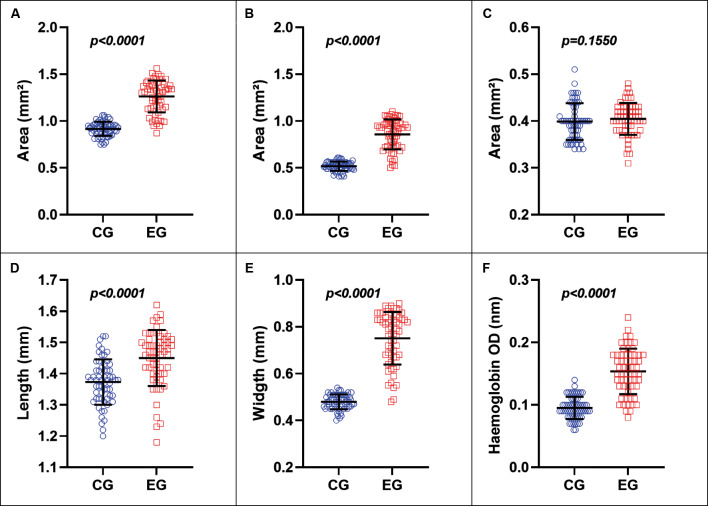
comparison of image body regions and haemoglobin (Hb) optical density (OD) (540 nm) parameters between *Lutzomyia longipalpis* females in the control group (CG, blue) and experimental group (EG, red). (A-B) Total body area (TBS) and abdominal area (ABS) (mm²); (C) TBS minus ABS area (mm²); (D-E) abdominal length (mm) and width (mm); (F) Hb OD (540 nm). Mann-Whitney statistical tests.

*Blood feeding-induced changes in body size* - Quantitative analysis of image-derived parameters from TBS (whole body excluding appendages and ABS (abdomen) ROIs revealed substantial morphological differences between unfed (CG) and blood-fed (EG) females (Fig. 3).

Blood-fed females displayed a larger mean area for TBS (an increase of 37.8%) and ABS (an increase of 65.9%) compared to the controls (Fig. 3A-B). However, when the difference between TBS and ABS areas was analysed, values were similar (1.4%) (Fig. 3C), suggesting that the overall increase was primarily attributable to abdominal expansion following blood ingestion. Differences in abdominal dimensions were markedly greater in width (an increase of 56.6%) compared to length (an increase of 5.6%) between the EG and CG groups (Fig. 3D-E). As abdominal expansion increases the width-to-length ratio as a proxy for abdominal distension, blood-fed females exhibited significantly higher “circularity” (0.71 ± 0.04 vs. 0.60 ± 0.03; Mann-Whitney test, p < 0.01) and “roundness” (0.52 ± 0.04 vs. 0.35 ± 0.03; Mann-Whitney test, p < 0.01) compared to controls. Moreover, a distinct reddish abdominal coloration (mean grey value 93.4 ± 11.00 vs 142.1 ± 10.36; Mann-Whitney test, p < 0.001) was observed in engorged females, contrasting with the brownish abdomen of unfed specimens. The abdominal expansion observed in fed females is related to blood intake; the mean OD value from the Hb measurements was 63.3% higher in the EG than in the CG (Fig. 3F).

**Fig. 4:**
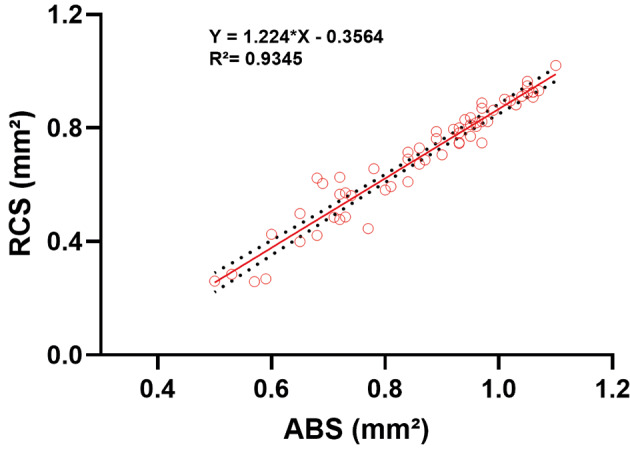
linear regression analysis was used to evaluate blood feeding in 59 females *Lutzomyia longipalpis* [experimental group (EG)] comparing the areas (mm²) of the red abdominal selection (RCS) with the abdominal region (ABS) obtained from image processing. Dashed lines indicate confidence interval.

*Analysis of blood-fed females based on abdominal colour* - To ensure consistency, one individual (the first from left to right in Fig. 2B) was identified as an outlier (absolute studentised residual > 3 and Cook’s distance > 4/n in both OD × ABS and OD × RCS regressions) and was excluded from subsequent analyses. This specimen’s OD value (0.15 at 540 nm) was much higher than expected given its very low ABS and RCS values (0.52 and 0.13 mm², respectively). This divergence may have been caused by pipetting or reading errors, leading to an overly high OD for this individual. Analysis of the remaining blood-fed females (EG; n = 59) showed that the red-coloured abdominal region (RCS; 0.70 ± 0.20 mm²) represented, on average, 79.54% of the total abdominal area (ABS; 0.86 ± 0.15 mm²), ranging from 45.30% to 92.98%. The RCS and ABS areas showed a strong positive correlation (R² = 0.93) (Fig. 4).

**Fig. 5:**
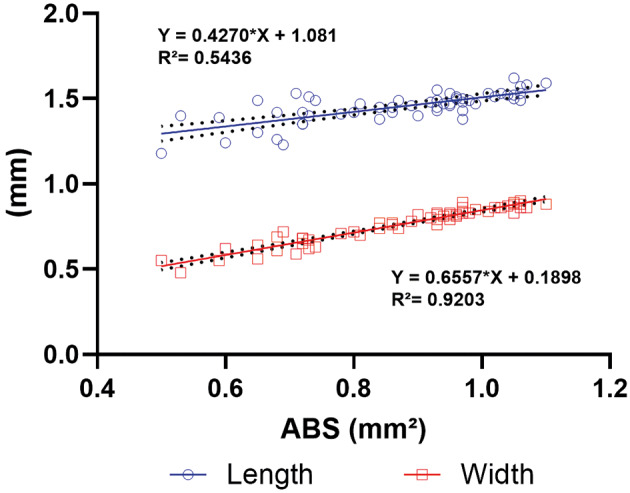
linear regression models relating width (red, mm) and length (blue, mm) of the abdominal region (ABS) to total area (mm²), obtained through image analysis of blood-fed of 59 female *Lutzomyia longipalpis* fed with blood [experimental group (EG)]. Abdominal width and length were estimated from the major (~length) and minor (~width) axes calculated by the Fit Ellipse tool. Dashed lines indicate confidence interval.

*Regression analysis of abdominal expansion parameters* - Since the abdomen’s lateral profile of insects (CG and EG) fitted an elliptical shape, the “Fit Ellipse” tool was employed to estimate the length (major axis) and width (minor axis) of the sand flies’ abdomens (Fig. 1H). Regression analysis indicated that abdominal expansion in blood-fed females was strongly associated with an increase in width (R² = 0.92) rather than length (R² = 0.54) (Fig. 5), highlighting width as the major contributor to abdominal growth.

**Fig. 6:**
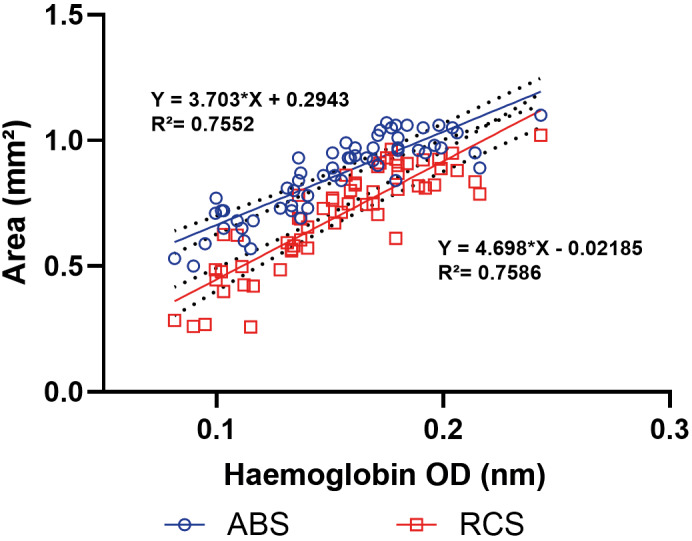
linear regression models for the area parameters (mm²) of the abdominal region (ABS) and the red-coloured abdominal selection (RCS) as functions of optical density (OD) (540 nm) values obtained from the haemoglobin (Hb) assay in 59 females *Lutzomyia longipalpis* [experimental group (EG)]. Dashed lines indicate confidence interval (CI).

*Correlation between Hb quantification and image-derived parameters* - Table presents a Pearson correlation analysis between Hb OD values and image-derived parameters from abdominal regions (ABS and RCS) of sand flies. Chosen from the parameters available in the “Set Measurements” of Fiji Imagej menu, those that reach correlations greater than ~0.7 in ABS or RCS regions. Two of these parameters (Area and Width) showed very strong correlations (R = 0.87) between blood meal size and the abdominal areas (ABS or RCS). In addition, the linear regression that best fit the relationship between Hb OD values and imaging abdominal characteristics of blood-fed females was obtained using ABS and RCS Areas (R² ~ 0.75) (Fig. 6).

**TABLE:** Pearson correlation coefficients between optical density(OD) (540 nm) values from the haemoglobin (Hb) assay and various image-derived parameters of abdominal selection (ABS) and red-coloured selection (RCS) of blood-fed *Lutzomyia longipalpis* females

Parameter	ROI	
	ABS (n = 59)	RCS (n = 59)
Area	0.87	0.87
Mean	0.02	0.23
Length (Major)	0.55	0.79
Width (Minor)	0.86	0.87
Circularity	0.66	0.15
IntDen	0.70	0.81
Round	0.72	0.33

ROI: region of interest.

## DISCUSSION

Phlebotomine sand flies are principal vectors of several medically important pathogens, including phleboviruses, bacteria, and protozoans such as *Leishmania*. Blood feeding is one key determinant of their reproductive success and vectorial capacity. This physiological process initiates a cascade of events relevant to host-parasite interactions and pathogen transmission. This study introduces and validates a novel image-based methodology to assess morphological changes induced by blood ingestion in *Lu. longipalpis* females, benchmarking its accuracy against standard biochemical Hb quantification.

Our findings reveal that blood-feeding elicits substantial and quantifiable morphological alterations, particularly in abdominal width. Engorged females exhibited a 56.6% increase in abdominal width, while abdominal length increased by only 5.6%. This asymmetrical expansion led to a pronounced change in body conformation, shifting towards a more rounded morphology, which was robustly captured through circularity and roundness metrics derived from lateral-view images. A markedly strong correlation (R² = 0.93) was observed between abdominal distension and the area of transilluminated reddish coloration, indicating that image-based analysis can serve as a reliable and non-invasive proxy for estimating blood meal volume. Remarkably, this approach enables the detection of blood-fed individuals even without visually obvious abdominal enlargement, addressing a critical limitation in conventional assessment methods that rely on gross visual inspection or gravimetric measurements.

Further supporting the validity of this approach, we found high correlations between Hb content and key morphometric parameters such as area (0.87 and 0.87) and width (0.86 and 0.87) for the ABS and RCS ROIs, respectively (Table). Conversely, the “mean grey value” parameter exhibited negligible correlation (ABS 0.02 and RCS 0.23), likely due to the two-dimensional constraints of the imaging process, interference from sclerotised abdominal structures, and the limited ability of grayscale intensity to reflect internal volume. While Hb quantification remains a sensitive benchmark for measuring blood intake, it is inherently destructive, chemically hazardous, and unsuitable for longitudinal studies. In contrast, the image-based technique described here is non-invasive, reusable, and cost-effective, offering a viable, especially powerful alternative when paired with open-source tools like Fiji/ImageJ. The gravimetric method, though widely used, also presents important constraints, particularly the inability to resolve individual blood intake levels in pooled or subtly engorged specimens without high-precision analytical balances. Our image-based approach effectively overcomes these limitations and introduces a practical solution for fine-scale phenotyping in vector biology studies.

Theoretically, this method is compatible with tracking live insects. This is important because many aspects of sand fly biology remain poorly understood - largely because, of the 927 known species, only 21 have established laboratory colonies.^40^ ^,^ ^41^ The development of non-destructive methodologies suitable for repeated measurements provides new opportunities to clarify various aspects of sand fly physiology, particularly those triggered by blood ingestion. Although studies have shown that sand flies anaesthetised with cold or CO₂^42^ ^,^ ^43^ ^,^ ^44^ ^,^ ^45^ can be used to initiate new colonies or to oviposit, the present study did not evaluate the recovery of individuals following cold anaesthesia at -20ºC.

In conclusion, our results demonstrate that image-based analysis constitutes a powerful, accessible, and scientifically rigorous tool for the indirect quantification of blood meals in *Lu. longipalpis*. We support the use of image and Hb-based approaches to enhance precision, reproducibility, and scope across diverse experimental frameworks for estimating blood meals in vector research.

## SUPPLEMENTARY MATERIALS

Supplementary material

## Data Availability

All relevant data have been included in the manuscript.
