# Supplementary material for: A non-destructive image-based approach to quantify blood meal size in Lutzomyia longipalpis (Diptera: Psychodidae)
Source: Mem Inst Oswaldo Cruz. 2026 May 1;121:e250158. doi: 10.1590/0074-02760250158 (PMC13143169; doi:10.1590/0074-02760250158)
Supplement: Supplementary material [file 1678-8060-mioc-121-e250158-s1.pdf]

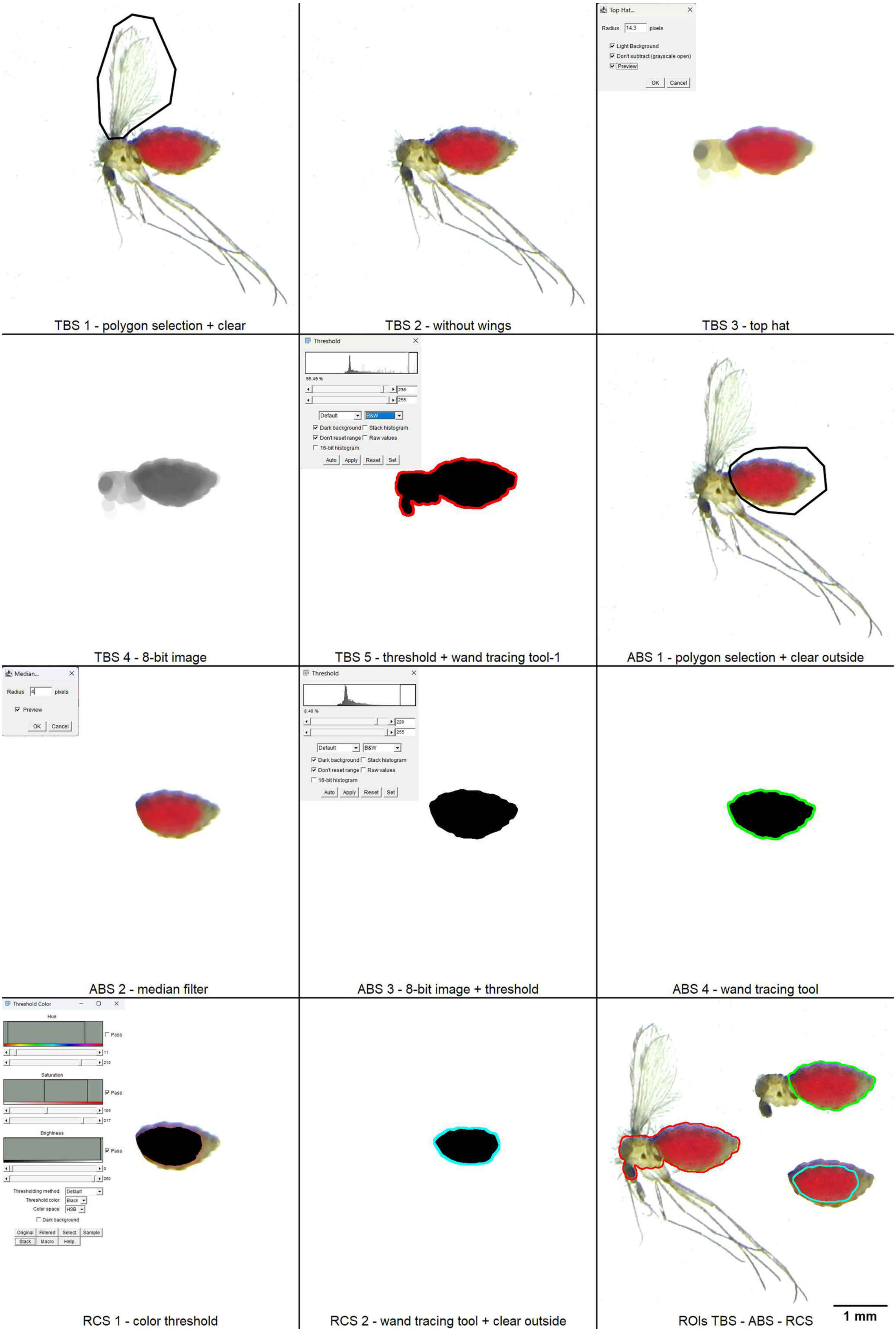

Steps in the segmentation process for obtaining regions of interest (TBS - ABS - RCS) in image analysis of female *Lutzomyia longipalpis*.
